# Engineered phosphorus dendrimers as powerful non-viral nanoplatforms for gene delivery: a great hope for the future of cancer therapeutics

**DOI:** 10.37349/etat.2022.00071

**Published:** 2022-02-25

**Authors:** Serge Mignani, Xiangyang Shi, Maria Bryszewska, Dzmitry Shcharbin, Jean-Pierre Majoral

**Affiliations:** 1 Université Paris Descartes, PRES Sorbonne Paris Cité, CNRS UMR 860, Laboratoire de Chimie et de Biochimie Pharmacologiques et Toxicologique, 45, rue des Saints Peres, 75006 Paris, France; 2 CQM-Centro de Química da Madeira, MMRG, Universidade da Madeira, Campus da Penteada, 9020-105 Funchal, Portugal; 3 College of Chemistry, Chemical Engineering and Biotechnology, Donghua University, Shanghai 201620, China; 4 Department of General Biophysics, Faculty of Biology and Environmental Protection, University of Lodz, 141/143 Pomorska St., 90-236 Lodz, Poland; 5 Institute of Biophysics and Cell Engineering of NASB, Akademicheskaya 27, 220072 Minsk, Belarus; 6 Laboratoire de Chimie de Coordination du CNRS, 205 route de Narbonne, 31077 Toulouse, Cedex 4, France; 7 Université Toulouse, 118 route de Narbonne, 31077 Toulouse, Cedex 4, France; Changchun Institute of Applied Chemistry, Chinese Academy of Sciences, China

**Keywords:** Phosphorus dendrimers, gene delivery, cancer therapeutics

## Abstract

During the past two decades, tremendous progress has been made in the dendrimer-based delivery of therapeutic molecules including, for instance, small molecules, macromolecules, and genes. This review deals with recent successes in the development of promising biocompatible phosphorus dendrimers, a specific type of dendrimer, to deliver genes to treat cancers.

## Introduction

In 2020, there were 19.3 million new cancer cases and almost 10.0 million cancer deaths. The ‘top five’ most prevalent cancers are breast (11.7%), lung (11.4%), colorectal (10.0%), prostate (7.3%), and stomach (5.6%) cancers. Lung cancer remains the leading cause of cancer death, with an estimated 1.8 million deaths (18%), followed by colorectal (9.4%), liver (8.3%), stomach (7.7%), and breast (6.9%) cancers [1]. According to the National Cancer Institute, by 2040, the number of new cancer cases is estimated to rise to about 30 million per year, with about 16.4 million deaths due to cancer [2]. Old-generation antineoplastic drugs (e.g., cisplatin, vincristine, etoposide, and cyclophosphamide) continue to be the important first-line ‘gold-standard’ treatment, but new-generation targeted therapies (e.g., MabThera, Iressa, Velcade, Glivec, Avastin, and Herceptin) are achieving blockbuster status and are displacing older drugs [3–6]. Control of the cell cycle, cell growth, and cell division allow scientists to narrow down the drug target development [7]. The growth of this new strategy is based on the delivery of selective therapeutics targeting cancer cells and encompassing therapeutic nucleic acids, including RNA interference (RNAi) effectors. The major developments have occurred with small interfering RNAs (siRNAs) and microRNAs (miRNAs) [8], against breast cancers for instance [9]. As therapeutics, the main limitation of their clinical development is their delivery and *in vivo* stability. When administered systemically, they are highly unstable and rapidly degraded in a biological environment (e.g., serum nucleases) due to their rapid metabolization by the body, which is armed with specific enzymes and many defense systems, including antibodies [10]. Lipid- and polymer-based nanoparticles as gene vectors, including siRNA and miRNA delivery systems, have been intensively developed for cancer therapy, to surmount these physiological barriers, e.g., to escape from endosomal degradation [11].

Nanomedicine is the medical application of nanotechnologies and covers all the techniques, tools, and processes that allow the manipulation of matter on a scale of fewer than one hundred nanometers. Nanomedicine involves the use of nanovectors to carry and release a drug in a very specific way into tissues and then into the target cells. Nanovectors are about 50–100 nm in size, that is, about 10 to 100 times smaller than a living cell. This nanoparticle vectorization increases the efficacy and bioavailability of drugs while reducing the dose and toxicity [12]. At present, a relatively large arsenal of precision nanoparticles has been designed and developed as vectors for drug delivery, for the application of nanotechnology in nanomedicine including PEGylated and non-PEGylated liposomes, polymeric nanoparticles such as dendrimers and polymer-drug conjugates, nanocrystals encompassing iron oxide, micelles, polymer-protein conjugates, and degradable nanogels [13, 14]. During the past two decades, tremendous progress has been made in the dendrimer-based delivery of therapeutic molecules including, for instance, small molecules and macromolecules [15], and genes [16, 17]. In this review, we survey recent successes in the development of promising biocompatible phosphorus dendrimers, a specific type of dendrimers, to deliver genes to treat cancers.

## Dendrimers as nanovectors in nanomedicine: a concise overview

**Figure 1.**
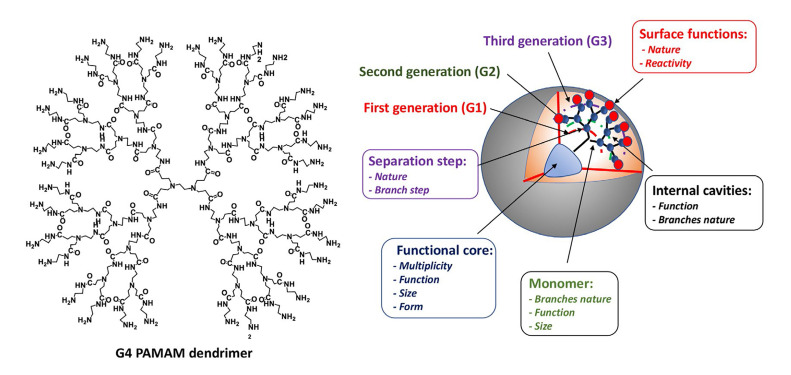
Chemical structure of 2D G4 PAMAM dendrimers and functional core, surface functions, internal cavities, and monomer elements as an example

Within the area of nanomedicine, dendrimers are well-defined and homogeneous artificial polymeric macromolecules, with nano-sized three-dimensional macromolecular architecture [18]. They have monodispersed structures. The word “dendrimer” comes from the Greek words “dendron”, meaning tree or branch, and “meros”, meaning part. The size and molecular weight can be fully controlled by the generation [e.g., generation 0 (G0)–Gn], where G0, G1, G2, and G3 refer to dendrimers with the zero, first, second, and third levels of branching, respectively. These nanoobjects are a few nanometers in size and are built by assembling stones, starting from a central point, called the “core”, and going three-dimensionally in different directions (radial layers) towards the outside of the macromolecule (repetitive units), implementing a large variety of cationic, anionic, or neutral chemical moieties on the surface to control their density and afford a tailored architecture, thereby enabling the fine-tuning of their physicochemical and/or biological properties. The connections between the different stones are made by branches that confer the cohesion and stability of the nanoobjects [19]. To date, there are over 100 dendrimer families. The commercially available poly(amidoamine) (PAMAM) dendrimers were first reported by Tomalia and Fréchet [20]. As shown in Figure 1, the 2D chemical structure of G4 PAMAM dendrimers and their different attributes, including functional core, surface functions, internal cavities, and monomer elements.

Several other dendrimers, such as poly(propyleneimine) (PPI), poly-L-lysine (PLL) scaffold dendrimers, poly-L-glutamic acid polyesters (PGLSA-OH), poly(2,2-bis(hydroxymethyl)propionic) acid (bis-MPA) scaffold dendrimers, glycodendrimers, carbosilane (CBS) dendrimers, and phosphorus dendrimers have also been studied for their role in drug delivery [21]. Importantly, biocompatible phosphorus dendrimers have been developed as drugs themselves [22]. Some examples were emphasized, except for PAMAM and phosphorus dendrimers. Regarding the phosphorus dendrimer types, the main therapeutic domains developed by Majoral et al. are as follows: anti-inflammatory [23], anti-tuberculosis [24], anti-Alzheimer’s disease [25], anti-prion [26], and anti-cancer [22, 27]. The theranostic realm in oncology has also been developed using phosphorus dendrimers and dendrons [28]. In the drug delivery realm, the main objective of dendrimer application is to improve the therapeutic outcomes of the loaded drugs, such as their pharmacokinetic (PK)/pharmacodynamic (PD) profiles. Biologically targeted active compounds, including small molecules, macromolecules, peptides, and metal nanoparticles, can be encapsulated inside the void spaces of dendrimers, whereas small molecules, macromolecules, targeting peptides, antibodies, and nucleic acids can be conjugated or complexed with adequate end surface groups.

Despite developing a strategy based on the chemical modification of the siRNA molecule to overcome the limitation of their instability, the rapid development of nanoparticle-mediated therapeutic nucleic acid delivery has been intensively investigated [29, 30]. The main challenge is the lack of an adequate intracellular delivery system to deliver exogenous genes into cells, which remains an important challenge in medicine. Safe dendrimers represent a powerful system to transport and deliver therapeutic nucleic acids into the cells based on their tailored physicochemical properties. The objective is to specifically suppress the expression of single or multiple targeted genes. Importantly, gene therapeutic tools based on RNAi provide a broader choice of target proteins in comparison, for instance, with classical therapeutic approaches. From a general perspective, numerous therapeutics using the two RNAi effectors siRNAs and miRNAs, which are the two major therapeutic nucleic acids used, are currently in the process of preclinical and clinical trials. Cationic polymers have been studied extensively as potential nucleic acid carriers within the framework of the delivery of genetic material at a post-transcriptional level of the disease-related gene [29, 30]. Specifically, as non-viral vectors [31], a wide range of safe and effective delivery polycationic dendrimer systems that can compact and efficiently deliver RNAi effectors have emerged as promising gene delivery systems. The major advantages of the non-viral delivery systems compared to viral vectors can be highlighted in their simplicity to synthesized, strong stability, tunable modifications, and biosafety profile. The major weaknesses of non-viral delivery systems are the necessity to overcome obstacles including effectively breaking through extra- and intra-cellular barriers, and avoiding immune defense mechanisms [32, 33].

In addition to cationic lipids [34], cationic peptides [35], and liposomes [36], the non-viral gene delivery dendrimer systems commonly developed include PAMAM, PPI, CBS, PLL, and phosphorus dendrimers. Based on the dynamic structure of histone, structurally flexible cationic amphiphilic PAMAM dendrimers were also developed for effective siRNA delivery and successful gene silencing [37, 38]. The electronegative nature of RNAi effectors such as siRNAs and miRNA allows their complexation with the polycationic dendrimer surface, affording a positively charged and stable siRNA/dendrimer complex (called a dendriplex) through electrostatic interactions, for effective cellular uptake to implement gene expression [39, 40]. Importantly, this construction is able to protect RNAi effectors from degradation, and the excess positive charges on the dendriplex promote penetration through cell membranes, generally by adsorption endocytosis [41]. Notably, the toxicity of cationic macromolecules in general, and polycationic dendrimers in particular, remains an acute problem. Nevertheless, the preparation and development of safe dendrimers have been highlighted, including phosphorus dendrimers for instance [42]. After being taken up by cells, the dendriplex is firstly located in endosomes. Then, in the late endosome or lysosome form, the rupture of the endosomal membrane occurs, due to the presence of the protonated dendrimers. Finally, during the endosomal escape, dendriplex disintegration takes place, and the dendriplex components, such as nucleic acids, are released into the cytosol, where the biological effects of RNAi occur [41]. A recent tutorial review from Tarach and Janaszewska [30] highlighted recent studies exploring the development of PAMAM dendrimers in anticancer gene therapies. Three different modification strategies have usually been used: 1) surface modification with the introduction of a functional group on the surface; 2) development of hybrid vectors by entrapment of gold nanoparticles; and 3) development of supramolecular self-assemblies for biomedical applications.

### Phosphorus dendrimers as non-viral gene delivery systems

**Figure 2.**
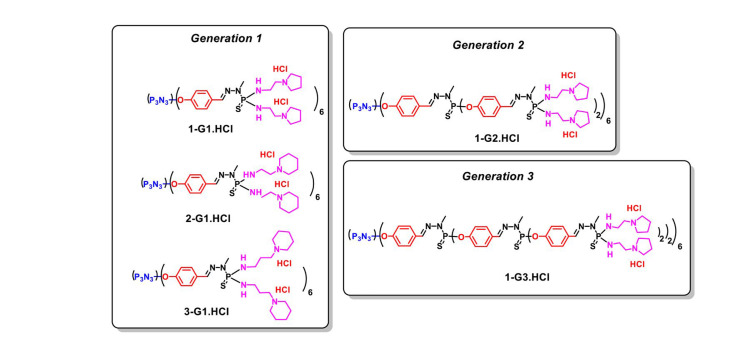
Chemical structure of 1-G1.HCl, 1-G2.HCl, 1-G3.HCl, 2-G1.HCl and 3-G1.HCl dendrimers

As shown in Figure 2, Chen et al. [43] designed, synthesized, and developed five original G1–G3 cationic phosphorus dendrimers bearing cyclic amine moieties, such as 1-(2-aminoethyl) pyrrolidine [hydrochloric acid (HCl); 1-G1.HCl, 1-G2.HCl, and 1-G3.HCl)], 1-(3-aminopropyl) piperidine (2-G1.HCl), or 1-(2-aminoethyl) piperidine (3-G1.HCl), on their surface for exogenous gene delivery toward cancer gene therapy applications. The gene was first complexed with the plasmid DNA (pDNA) encoding enhanced green fluorescent protein (EGFP) as a simple model to test the gene compaction ability of phosphorus dendrimers. The cytotoxicity profile of the dendrimer/pDNA polyplexes was analyzed, along with the gene delivery efficiency. All the prepared dendrimer/pDNA polyplexes demonstrated good cytocompatibility against the human cervical carcinoma cell line (HeLa cells) via the cholecystokinin-8 (CCK-8) viability assay; interestingly, the dendriplex with the 1-(2-aminoethyl) pyrrolidine group (1-G1.HCl) displayed the most effective gene delivery efficiency to HeLa cells, as confirmed by flow cytometry and fluorescence microscopic imaging analyses. The cell viability was at least greater than 50%, with the highest concentration of polyplexes of 3,000 nmol/L and with a N/P ratio (molar ratio of the positive charge of the dendrimers to phosphates in the pDNA backbone) of 20. The cell viability decreased in a concentration-dependent manner. It is known that the hydrodynamic size and surface potential of the polyplexes are critical parameters for high-performance gene delivery. Different N/P ratios were used, such as 10–30. The DNA compaction ability followed the order: 1-G3. HCl (N/P = 0.5 or higher) > 1-G1.HCl (N/P = 1 or higher) > 1-G2.HCl (N/P = 2 or higher).

**Figure 3.**
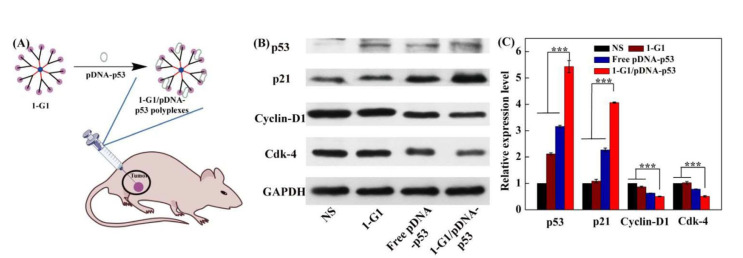
(A) Formation of 1-G1.HCl/pDNA-p53 polyplexes for *in vivo* gene delivery experiments; (B) western blot assay indicating the expression of the proteins related to G0/G1 phase in xenografted HeLa tumor cells at 4 days post-treatment of normal saline (NS), 1-G1.HCl, free pDNA-p53, and 1-G1.HCl/pDNA-p53 polyplexes (20 μg pDNA/mouse for pDNA groups). The glyceraldehyde-3-phosphate dehydrogenase (GAPDH) protein was used as an internal control; (C) quantitative analysis of the G0/G1 phase-related protein expression level from the western blot data *in vivo* *Note*. Adapted from “Revisiting cationic phosphorus dendrimers as a nonviral vector for optimized gene delivery toward cancer therapy applications,” by Chen L, Li J, Fan Y, Qiu J, Cao L, Laurent R, et al. Biomacromolecules. 2020;21:2502–11 (https://pubs.acs.org/doi/10.1021/acs.biomac.0c00458). Copyright 2020, American Chemical Society.

The 1-G1.HCl dendrimers were then used to vectorize pDNA encoding both EGFP and tumor protein 53 (p53) protein for cancer gene therapy applications, as shown in Figure 3. Interestingly, cell cycle arrest and western blotting studies showed the regulation of the cyclin-dependent kinase inhibitor (p21) and cyclin-dependent kinase 4 (Cdk-4)/Cyclin-D1 expression, causing cancer cell apoptosis. Importantly, powerful intracellular gene delivery was fully validated *in vivo* in a xenografted tumor model after intratumoral injection, without systemic toxicity. Based on the western blot assay of the protein expression, 1-G1.HCl/pDNA-p53 polyplexes showed the strongest p53 and p21 protein expression inducing apparent G1/S cell cycle arrest checkpoint, and lower Cdk-4 and Cyclin-D1 expression compared with 1-G1.HCl and free pDNA-P53. These results are fully consistent with the *in vitro* gene delivery data (vide supra).

**Figure 4.**
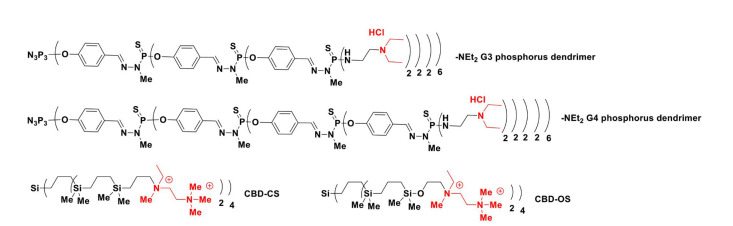
Chemical structure of AE2G3 (AE2G3.HCl) and G4 (AE2G4.HCl) phosphorus dendrimers

Notably, in early studies, Ihnatsyeu-Kachan et al. [44] explored the multi-target cytotoxic effects of an original combination system of siRNA cocktails complexed with G3–4 polycationic phosphorus dendrimers in combination with the therapeutic anticancer agent 5-fluorouracil (5-FU) against the HeLa cells. The siRNAs, such as si B cell leukemia/lymphoma-2 (siBCL-2), si B-cell lymphoma-extra large (siBCL-xL), and si myeloid cell leukemia-1 (siMCL-1), downregulate the anti-apoptotic gene BCL-2 family, including *BCL-xL*, *BCL-2*, and *MCL-1*. These genes regulate cell death, induce the suppression of cell apoptosis, and are overexpressed in many types of cancer, including ovary, breast, pancreas, acute myeloid leukemia, and skin cancers. The objective was to analyze the enhancement of the anticancer activity of this chemotherapeutic system *versus* each component alone. As shown in Figure 4, the cationic G3 (AE2G3, AE2G3.HCl) and G4 (AE2G4.HCl) phosphorus dendrimers have 48 and 96 protonated piperidinium groups on their surface, respectively, to compact siRNAs and showed efficacy to treat HeLa cells with half-maximal inhibitory concentration (IC_50 _) values of approximately 1–3 μmol/L (72 h). These polycationic phosphorus dendrimers provided a high ability to stabilize and to complex pro-apoptotic siRNAs and provided 80–100% siRNA uptake in HeLa cells. Interestingly, the viability of HeLa cells treated with the AE2G3-based pro-apoptotic siRNAs dendriplexes (dendrimer/siRNA charge ratio 10:1) decreased with the increasing level of pro-apoptotic AE2G3 siRNA: ~70% with 25 nmol/L of siRNA, and almost complete cell death in low concentrations (50 nmol/L and 100 nmol/L) of the AE2G3 siRNA cocktail. In HeLa cells, the cytotoxic effect of AE2G3-based dendriplexes was significantly higher (> 10 times) than that of the AE2G4-based dendriplexes (charge ratio 10:1). A considerable increase of 5-FU cytotoxic effects were observed with the addition of AE2G3/siRNA cocktail complexes (charge ratios: 5/1 and 10:1) in low doses (25 nmol/L of pro-apoptotic siRNAs and 25 μmol/L of 5-FU).

**Figure 5.**
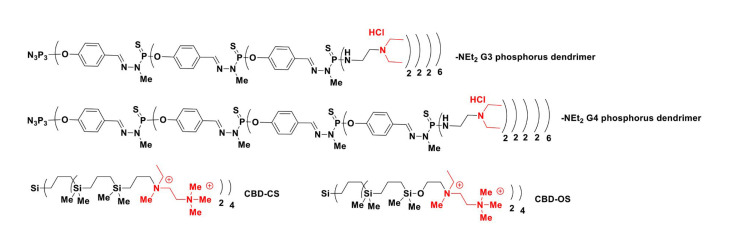
2D chemical structures of G3 and G4 phosphorus dendrimers with -NEt_2_ groups on the surface and CBD-CS and CBD-OS

Ionov et al. [45] presented an interesting and noteworthy study exploring newly engineered nanomaterials as non-viral carriers to deliver a cocktail of genes based on three polycationic dendrimer types, including G3–4 protonated PAMAM dendrimers (G3: 32 protonated amino groups on the surface; G4: 64 protonated amino groups on the surface, Figure 1), G3–4 protonated phosphorus dendrimers (G3: 48 NEt.HCl groups on the surface, G4: 96 NEt.HCl groups on the surface), and two G2 polycationic carbosilane dendrimers named CBD-OS bearing 8 NMe(Et)^(+)^-CH_2_-CH_2_-N(Me)_3 _^(+)^ groups on the surface and its stable analog named CBD-CS bearing also 8 Me(Et)^(+)^-CH_2_-CH_2_-N(Me)_3 _^(+)^ groups on the surface for cancer therapy [45] (Figure 5).

**Figure 6.**
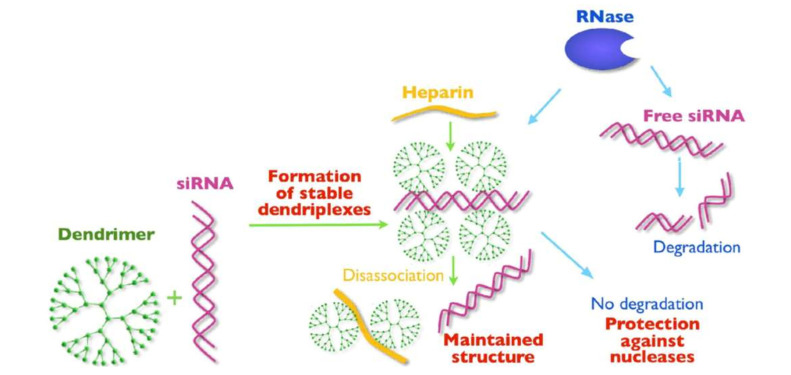
Representation of interactions between dendrimers and siRNA and protective effects against RNase degradation [45] *Note*. Reprinted from “Anticancer siRNA cocktails as a novel tool to treat cancer cells. Part (A). Mechanisms of interaction,” by Ionov M, Lazniewska J, Dzmitruk V, Halets I, Loznikova S, Novopashina D, et al. Int J Pharm. 2015;485:261–9 (https://doi.org/10.1016/j.ijpharm.2015.03.024). Copyright 2015, Elsevier.

These three dendrimer types were compacted with a cocktail of anticancer siRNAs (siBCL-xL, siBCL-2, siMCL-1). The dendrimer/siRNA dendriplexes were characterized by techniques including fluorescence, zeta potential, dynamic light scattering, circular dichroism, gel electrophoresis, and transmission electron microscopy. All the dendrimers complexed with siRNAs and the oligoribonucleotides were released from the dendriplexes by the action of heparin. Based on gel electrophoresis assays, the dendrimers were also effective in protecting siRNA from RNase A activity. A schematic description of the interactions between the dendrimers and siRNA, as well as the protective properties of the dendrimers against RNase degradation, are shown in Figure 6.

**Figure 7.**
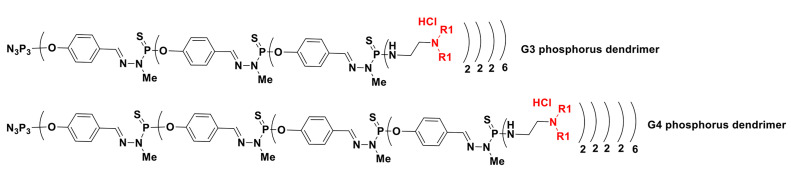
Chemical structure of G3 and G4 phosphorus dendrimers corresponding to Table 1

Further work by the same team reported the analysis of the transfection of these dendriplexes bearing the cocktail of siRNAs (siBCL-xl, siBCL-2, and siMCL-1, vide supra) *versus* single siRNAs in HeLa and human leukemia HL-60 cells [46]. Cocktails were more effective compared with single siRNA, allowing the concentration of siRNAs to be decreased in cell treatment. The dendrimers were then compared as siRNA carriers, the most effective being the phosphorus-based dendrimers, although they were also the most cytotoxic. Taken together, the possible application of dendrimers in anticancer gene therapy can be highlighted as follows: 1) construction a: G4 phosphorus dendrimer plus 250 nmol/L siRNA cocktails showed efficient cellular uptake but a high cytotoxic effect; 2) construction b: G3 phosphorus dendrimer, G4 PAMAM dendrimer, or CBD-CS CBS dendrimer plus 100 nmol/L siRNA cocktails displayed moderate cellular uptake; and 3) construction c: G3 PAMAM dendrimer or CBD-OSi CBS dendrimer plus 250 nmol/L siRNA cocktails exhibited a low cytotoxic effect. Regarding the potential therapeutic applications, construction a should be developed for emergency treatment of the last stages of cancers (like chemotherapy), construction b should be developed for the treatment of the middle stages of cancer (safer than chemotherapy), and construction c should be developed for the long-term treatment of early-stage cancer (similar to the permanent insulin treatment of diabetes). Additional phosphorus dendrimers used for gene delivery of siRNAs were highlighted in Figure 7 and Table 1.

**Table 1.** Additional phosphorus dendrimers used for gene delivery of siRNAs corresponding to Figure 7

**Construction**	**R1**	**Studies**	**Reference**
Plasmid and oligonucleotides	-Et	DNA delivery in HeLa and NIH 3T3 cells	[47]
Anti-HIV siRNA		siP24 delivery in HEK293 cells	[48]
siRNA (siPLK1)		Delivery in MDA-MB-231 (triple-negative breast cancer) and MCF-7 cells	[49]
Plasmid**:** green fluorescent protein (*GFP*) reporter gene		Transfection of human stromal/mesenchymal stem cells	[50]
G4 phosphorus dendrimer			
Anti-TNFα siRNA	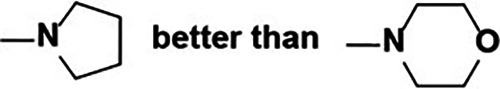	*In vivo* via nasal administration	[51]

HIV: human immunodeficiency virus; PLK1: polo-like kinase 1; TNFα: tumor necrosis factor alpha

## Conclusions and perspectives

During the past two decades, immense progress has been made in the dendrimer-based delivery of genetic materials to treat cancers. Nowadays, the siRNA cancer therapeutics field represents an effective strategy to reduce disease progression and to cure disorders where conventional therapeutics are non-effective, allowing hope due to its ability to repress the translation of any disease-causing protein via gene silencing. Within the nanomedicine realm, the main challenge is to develop safe and effective synthetic nanocarriers of genetic material encompassing siRNAs and miRNAs that are non-toxic to normal cells and deliver the genetic materials specifically to the tumor cells. Dendrimers are promising nanocarriers due to their three-dimensional highly branched nano-sized structure (1–10 nm), high structural homogeneity, and unique physicochemical properties. Within the 100 families of dendrimers that have been developed, phosphorus dendrimers were very quickly developed as drugs themselves (a less trodden therapeutic realm) and also for gene therapy. They can be used as nanocarriers of active principles, for example, small molecules, peptides, siRNAs, mRNAs, etc., or as active principles by themselves. In the first case, the objective is to improve the PK and PD characteristics of the transported active molecule, whereas the second strategy is based on the intrinsic activity of the dendrimer, which is considered as a biologically active macromolecule. The main advantages of phosphorus dendrimers are as follows: 1) improvement of the PK/PD of encapsulated/complexed/conjugated drugs; 2) several possible routes of administration [e.g., oral, intravenous (i.v.), intradermal, intranasal, pulmonary]; 3) decrease of the systemic toxicity of the complexed/conjugated drugs, the ‘Trojan horse’ approach, to qualify the carried drug as best-in-class; and 4) several biological target types: organs, tissues, cells, and nucleus. The main biophysical attributes of phosphorus dendrimers are 1) their biocompatibility, without the introduction of a polyethylene glycol (PEG) chain; 2) their easy surface functionalization for therapy and imaging purposes (theranostic aspect already developed); 3) their monodispersity; and 4) their precisely controllable dimensions and architectures. In this short review, we have advocated and analyzed the development of phosphorus dendrimers as nanocarriers of siRNAs alone, or in a cocktail strategy, to treat cancers. Collectively, the results clearly show the very high potential of phosphorus dendrimers in gene therapy. Importantly, this precision cancer therapy, using non-viral carriers of genetic material, could strongly benefit from the combined use of cancer therapies with immune checkpoint blockade, as well as chimeric antigens receptor-T (CAR-T) cell strategies, allowing personalized medicine and the fine-tuning of a patient’s therapeutic response inducing the elimination of cancer cells. Importantly, based on the promising clinical trial successes by Starpharma (Australia) regarding the development of PEGylated poly-L-lysine dendrimer conjugating drugs such as docetaxel or irinotecan (phase II clinical trials) within the Dendrimer Enhanced Product (DEP^®^) platform, as well as the development of the polyanionic dendrimer named VivaGel^®^ as a microbicide for the prevention of HIV and herpes simplex virus (HSV) infections, it seems possible that the regulatory authorities [Food and Drug Administration (FDA) and European Medicines Agency (EMA)] can be convinced about the development of biocompatible dendrimers. These dendrimers must be produced under Good Manufacturing Practice (GMP) grade. One of the important challenges is to harmonize the criteria and guidelines facilitating the translation from the pre-clinical to the clinical phase. With this in mind, we have analyzed the guidelines in-depth and spurred the development of dendrimers in clinical trials [52, 53]. Strongly, we suggest developing the low generation of phosphorus dendrimers (G0 and G1) designed by Majoral and Mignani et al. [24] and highlighted as anti-tubercular agents. These low generation of phosphorus dendrimers have the following advantages *versus* high generation of phosphorus dendrimers: 1) few synthesis steps leading to low generation of phosphorus dendrimers with high yields; 2) high solubility in the water allowing numerous routes of administration (e.g., i.v., oral, nasal, pulmonary) and reproducible *in vitro* and *in vivo* biological activities; 3) high chemical stability for more than one year without noticeable degradation; 4) perfect reproducibility of the syntheses; 5) possibility to be prepared in large quantities under GMP conditions; 6) easy modification of the chemical nature and number of end groups on their surface-based on dendritic effect; and 7) inexpensive starting materials. These characteristics are to be added to the global profile of phosphorus dendrimers analyzed previously [42].

## Abbreviations

5-FU: 5-fluorouracil
AE2G3: cationic G3
BCL-2: B cell leukemia/lymphoma-2
BCL-xL: B-cell lymphoma-extra large
CBS: carbosilane
Cdk-4: cyclin-dependent kinase 4
G0: generation 0
MCL-1: myeloid cell leukemia-1
miRNAs: microRNAs
N/P: molar ratio of the positive charge of the dendrimers to phosphates in the plasmid DNA backbone
p53: tumor protein 53
PAMAM: poly(amidoamine)
PD: pharmacodynamic
pDNA: plasmid DNA
PK: pharmacokinetic
RNAi: RNA interference
siRNAs: small interfering RNAs

## Declarations

### Copyright

© The Author(s) 2022.
